# Silencing VEGFR-2 Hampers Odontoblastic Differentiation of Dental Pulp Stem Cells

**DOI:** 10.3389/fcell.2021.665886

**Published:** 2021-06-25

**Authors:** Kajohnkiart Janebodin, Rakchanok Chavanachat, Aislinn Hays, Morayma Reyes Gil

**Affiliations:** ^1^Department of Pathology, University of Washington, Seattle, WA, United States; ^2^Institute for Stem Cell and Regenerative Medicine, University of Washington, Seattle, WA, United States; ^3^Department of Anatomy, Faculty of Dentistry, Mahidol University, Bangkok, Thailand; ^4^Department of Bioengineering, University of Washington, Seattle, WA, United States; ^5^Albert Einstein College of Medicine, Montefiore Medical Center, Bronx, NY, United States

**Keywords:** VEGFR-2, dental pulp stem cells, tooth development, tooth regeneration, dental pulp, angiogenesis, VEGF, odontoblasts

## Abstract

Dental pulp stem cells (DPSCs) are a source of postnatal stem cells essential for maintenance and regeneration of dentin and pulp tissues. Previous *in vivo* transplantation studies have shown that DPSCs are able to give rise to odontoblast-like cells, form dentin/pulp-like structures, and induce blood vessel formation. Importantly, dentin formation is closely associated to blood vessels. We have previously demonstrated that DPSC-induced angiogenesis is VEGFR-2-dependent. VEGFR-2 may play an important role in odontoblast differentiation of DPSCs, tooth formation and regeneration. Nevertheless, the role of VEGFR-2 signaling in odontoblast differentiation of DPSCs is still not well understood. Thus, in this study we aimed to determine the role of VEGFR-2 in odontoblast differentiation of DPSCs by knocking down the expression of VEGFR-2 in DPSCs and studying their odontoblast differentiation capacity *in vitro* and *in vivo*. Isolation and characterization of murine DPSCs was performed as previously described. DPSCs were induced by VEGFR-2 shRNA viral vectors transfection (MOI = 10:1) to silence the expression of VEGFR-2. The GFP+ expression in CopGFP DPSCs was used as a surrogate to measure the efficiency of transfection and verification that the viral vector does not affect the expression of VEGFR-2. The efficiency of viral transfection was shown by significant reduction in the levels of VEGFR-2 based on the Q-RT-PCR and immunofluorescence in VEGFR-2 knockdown DPSCs, compared to normal DPSCs. VEGFR-2 shRNA DPSCs expressed not only very low level of VEGFR-2, but also that of its ligand, VEGF-A, compared to CopGFP DPSCs in both transcriptional and translational levels. *In vitro* differentiation of DPSCs in osteo-odontogenic media supplemented with BMP-2 (100 ng/ml) for 21 days demonstrated that CopGFP DPSCs, but not VEGFR-2 shRNA DPSCs, were positive for alkaline phosphatase (ALP) staining and formed mineralized nodules demonstrated by positive Alizarin Red S staining. The expression levels of dentin matrix proteins, dentin matrix protein-1 (*Dmp1*), dentin sialoprotein (*Dspp*), and bone sialoprotein (*Bsp*), were also up-regulated in differentiated CopGFP DPSCs, compared to those in VEGFR-2 shRNA DPSCs, suggesting an impairment of odontoblast differentiation in VEGFR-2 shRNA DPSCs. *In vivo* subcutaneous transplantation of DPSCs with hydroxyapatite (HAp/TCP) for 5 weeks demonstrated that CopGFP DPSCs were able to differentiate into elongated and polarized odontoblast-like cells forming loose connective tissue resembling pulp-like structures with abundant blood vessels, as demonstrated by H&E, Alizarin Red S, and dentin matrix staining. On the other hand, in VEGFR-2 shRNA DPSC transplants, odontoblast-like cells were not observed. Collagen fibers were seen in replacement of dentin/pulp-like structures. These results indicate that VEGFR-2 may play an important role in dentin regeneration and highlight the potential of VEGFR-2 modulation to enhance dentin regeneration and tissue engineering as a promising clinical application.

## Introduction

Dental pulp stem cells (DPSCs), a type of neural crest derived mesenchymal stem cells, have been isolated from the dental pulp, a loose connective tissue located in the center innermost part of tooth structure (Janebodin et al., 2011). Compare to other types of stem cells, this dental tissue-derived stem cell is considered a promising source of adult stem cells for regenerative dentistry and medicine given its accessibility, tremendous expansion and differentiation capacity. Isolation of DPSCs is a less invasive method compared to other sources of mesenchymal stem cells. DPSCs can be generated from various sources of dental tissues considered biological wastes such as wisdom teeth, embedded teeth, supernumerary teeth and inflamed teeth (Gronthos et al., 2000; Huang et al., 2008; Tomasello et al., 2017; Paz et al., 2018). Additionally, several studies have demonstrated its potential in tissue regeneration both *in vitro* and *in vivo* animal models (Gronthos et al., 2002; Victor and Reiter, 2017; Kabatas et al., 2018; Yamada et al., 2019; Sui et al., 2020).

We and others have shown that DPSCs exhibited *in vitro* multipotential capacity by differentiating to a variety of cell types such as osteoblasts, chondrocytes, adipocytes, neurons and smooth muscle cells (Gronthos et al., 2000; Janebodin et al., 2011; Shi et al., 2020). Moreover, *in vivo* studies revealed that DPSC transplants exhibit versatile ability for tissue regeneration in various specific conditions and animal models (Kerkis et al., 2008; Martinez-Sarra et al., 2017; Fernandes et al., 2018; Ullah et al., 2018). Research interest in DPSCs has increased with a hope to manipulate this stem cell for potential future clinical applications relating to both dental and non-dental tissue regeneration and diseases (Paz et al., 2018; Yamada et al., 2019).

The potential of DPSCs for regeneration purposes is not only based on their intrinsic differentiation capacity, but also their paracrine function such as c, neurotrophic, and immunomodulating abilities (Bronckaers et al., 2013; Luo et al., 2018; Andrukhov et al., 2019). Vascular endothelial growth factor (VEGF) signaling has been shown important for DPSCs-induced angiogenesis (Janebodin et al., 2013). Recently, VEGF has been shown to be important for the development and repair of skeletal tissue such as bone and cartilage (Hu and Olsen, 2016b). Osteogenic cells and hypertrophic chondrocytes express high levels of VEGF during intramembranous and intracartilaginous bone formation, respectively (Jacobsen et al., 2008; Berendsen and Olsen, 2014; Duan et al., 2016). In addition, several studies suggest that VEGF can stimulate osteoblast differentiation (Hu and Olsen, 2016a). Dysfunction of VEGF signaling results in defective osteoblast differentiation and osteogenesis (Liu et al., 2012). VEGF is notably expressed in multiple cell types including endothelial cells, osteoblasts, bone marrow and dental mesenchymal stem cells (Liu et al., 2012; Janebodin et al., 2013; Buettmann et al., 2019).

Dental pulp stem cells are important for dentin and tooth regeneration. Previous *in vivo* transplantation studies have shown that DPSCs were able to give rise to odontoblast-like cells (OLCs), form dentin/pulp-like structures, and induce blood vessel formation. Dentin formation was also found closely related to blood vessels (Janebodin et al., 2011). We have previously shown that DPSC-induced angiogenesis is VEGFR-2-dependent (Janebodin et al., 2013). However, the role of VEGFR-2 signaling in odontoblast differentiation of DPSCs is still not well understood.

In this present study, we aimed to investigate the role of VEGFR-2 signaling for odontoblastic differentiation of DPSCs. Murine DPSCs were isolated and well characterized from neonatal dental pulp tissue of first and second mandibular molar teeth. DPSCs were expanded and the expression of VEGFR-2 was silenced using a specific VEGFR-2 shRNA viral vector. The efficiency of silencing was evaluated in VEGFR-2 shRNA treated DPSCs compared to the control DPSCs transfected by CopGFP shRNA by determining both transcriptional and translational levels. Afterward, the odontogenic differentiation capacity of DPSCs in these two groups was examined both *in vitro* and *in vivo*.

## Materials and Methods

### Mouse Housing and Husbandry

All mouse experiments were performed in accordance with approved Institutional Animal Care and Use Committee (IACUC) guidelines, University of Washington. Mice were originally purchased from the Jackson Laboratory. Mice were housed in a specific-pathogen-free (SPF) environment in ventilated cages with filter tops (Allentown Inc., Allentown, NJ, United States). A maximum of 5 mice were housed together in the same cage. Mice were maintained in standard light/dark cycle (6:00 a.m. lights on, 6:00 p.m. lights off), standard food (5058 mouse breeding food), standard temperature (68–79°F), and no environmental restriction. Mice were allocated randomly to control and experimental groups.

### Isolation, Culture and Characterization of Murine DPSCs

Dental pulp tissues were dissected and pooled from 5-day-old neonatal murine teeth. DPSCs were isolated, cultured, expanded, and characterized as previously described (Janebodin et al., 2011). Briefly, dental pulp from lower molar teeth was gently isolated and kept in stem cell media described below. The tissue was washed with phosphate buffer saline (PBS) (HyClone). To release cells, the extracellular matrix was digested with Dispase II (1.2 units/ml), Collagenase IV (2 mg/ml) (Worthington) supplemented with CaCl_2_ (2 mM) in PBS for 1 h at 37°C. Then, an equal volume of stem cell media was added to the digested tissue prior to filtering through 70 mm nylon cell strainers (BD Falcon) and centrifuging at 300 × *g* for 10 min at room temperature. Cells were subsequently resuspended in stem cell media and single cell suspensions were plated at a density of 1000 cells/cm^2^.

Cells were cultured at 37°C under 5% O_2_ and 5% CO_2_ in stem cell media, containing a final concentration of 60% low-glucose DMEM (Gibco, Invitrogen), 40% MCDB201 (Sigma), 2% fetal calf serum (HyClone), insulin-transferrin-selenium (ITS) (Sigma), linoleic acid with bovine serum albumin (LA-BSA) (Sigma), 10^–9^ M dexamethasone (Sigma), 10^–4^ M ascorbic acid 2-phosphate (Sigma), 100 units/ml penicillin with 100 mg/ml streptomycin (HyClone), and 1 × 10^3^ units/ml leukemia-inhibitory factor (LIF-ESGRO, Millipore), 10 ng/mL EGF (Sigma) and 10 ng/mL PDGF-BB (R&D) (Breyer et al., 2006). Once more than 50% cell confluent, they were detached with 0.25% trypsin-EDTA (Invitrogen) and replated at a 1:4 dilution under the same culture condition with fresh media. Differentiation capacity of cultured cells was determined by *in vitro* multi-differentiation to bone, cartilage and fat cells as previously described (Gronthos et al., 1994; Gregoire, 2001; Dahlin et al., 2014). The osteo-odontogenic media contains serum-free media with 10% FBS, 10 mM β-glycerophosphate, 0.2 mM L-ascorbic acid, and 100 nM dexamethasone. The chondrogenic media contains serum-free high-glucose DMEM, 1% ITS+ premix (BD Biosciences), 50 mg/mL ascorbic acid, 100 nM dexamethasone, supplemented with 10 ng/ml TGF-β3 (Shenandoah Biotech). The adipogenic media contains serum-free media supplemented with 10% horse serum, 100 μM indomethacin (Alfa Aesar), 0.5 mM 3-isobutyl-1-methyl-xanthine (ACROS) and 1 μM dexamethasone.

### Silencing VEGFR-2 in Murine DPSCs

Murine DPSCs were stably transfected with VEGFR-2 shRNA to silence the expression of VEGFR-2 as per manufacturer’s protocol. Briefly, cells (1000 cells/cm^2^) were plated and cultured in stem cell media for 24 h. DPSCs were allowed to proliferate until 70% confluent before viral transfection. Then, cells were cultured in stem cell media with polybrene (5 μg/ml) to increase binding between the pseudoviral capsid and the cell membrane. Cells were infected by the VEGFR-2 shRNA lentiviral particles (Santa Cruz Biotechnology, Santa Cruz, CA, United States) with 10:1 of multiplicity of infection (MOI), then mixed gently, and incubated overnight. CopGFP control lentiviral particles (Santa Cruz Biotechnology) were also used as control to evaluate transduction efficiency at the similar MOI used in VEGFR-2 shRNA lentiviral particles. After overnight transduction, infected cells were replaced with stem cell media without polybrene and incubated overnight. To select stable infected cells expressing the shRNA, we expanded cells one more time in stem cell media overnight, and then selected them by exposure to puromycin dihydrochloride (5 μg/ml) (Sigma). The infected cells were cultured in puromycin-containing stem cell media until the resistant cells were identified. The homogeneous population of resistant cells was observed by the expression of Green Fluorescence Protein (GFP) in cells transduced with CopGFP control lentiviral particles. To determine the knockdown efficacy, the level of VEGFR-2 and VEGF-A in both silencing and control groups was determined by mRNA and protein levels through real-time PCR and immunofluorescence analyses.

### Immunofluorescence

Cells were fixed with 4% formaldehyde/PBS for 5 min, washed with 1% BSA in 0.1% Triton-X 100/PBS, and stained with primary antibodies as described in Supplementary Table 1 incubated overnight at 4°C. Goat-derived Alexa 594-conjugated secondary antibodies (Invitrogen) were diluted at 1:800 and incubated for 1 h. Cells were stained with 4′, 6-diamine-2-phenylindol (DAPI) at 1:1000 to visualize the nuclei. All antibodies were diluted in 1% BSA in 0.1% Triton-X 100/PBS. We used IgG isotype from the species made for the primary antibody (0.1 ug/ml) (Vector Laboratories Inc., Burlingame, CA, United States), were included for all staining.

### *In vitro* Osteo-Odontogenic Differentiation

VEGFR-2 shRNA treated DPSCs and CopGFP treated DPSCs (2 × 10^4^ cells/cm^2^, *n* = 4 wells/group) were incubated overnight in stem cell media at 37°C under 5% O_2_ and 5% CO_2_. After 24 h, each cell type was separately cultured in BMP-2 media, β-glycerophosphate plus ascorbic acid (BGP + Vit. C) media, VEGF media for 21 days to evaluate the osteo-odontogenic differentiation capacity with media change every 3 days. BMP-2 media is serum-free media containing 10% FBS, 100 ng/ml BMP-2 (Shenandoah Biotech), 0.2 mM L-ascorbic acid, and 100 nM dexamethasone (Hu et al., 2004). BGP + Vit. C media is serum-free media containing 10% FBS, 10 mM β-glycerophosphate (CalBiochem), 0.2 mM L-ascorbic acid, and 100 nM dexamethasone (Song et al., 2009). VEGF media is serum-free media containing 10 ng/ml VEGF (R&D) (Forgues et al., 2019). Serum-free media is 60% low-glucose DMEM, 40% MCDB201, ITS, LA-BSA, 10^–9^ M dexamethasone, 10^–4^ M ascorbic acid 2-phosphate, 100 units/ml penicillin with 100 mg/ml streptomycin. After differentiation, both cell groups were separately divided into 2 subgroups for RNA collection and staining.

### Alkaline Phosphatase Staining

After differentiation, cultured cells were stained by ALP staining kit (BioVision) as per manufacturer’s protocol. Briefly, the media were carefully removed from the cultured cell wells. Cells were fixed with 4% formaldehyde/PBS for 15 min at room temperature. Then, 500 μl of wash buffer was gently added and carefully removed using a pipette. 250 μl of ALP Staining Reagent solution was carefully added to completely cover the cells in each well of a 24-well plate. Cells were incubated for 30 min at 37°C before washed gently with 500 μl of wash buffer for 3 times. 300 μl of wash buffer was added and stained cells were imaged using a light microscope.

### Alizarin Red S Staining

Cultured cells were stained by Alizarin Red S staining kit (ScienCell Research Laboratories) as per manufacturer’s protocol. Briefly, the media were carefully removed from the cultured cell wells and cells were washed three times with PBS. Cells were fixed with 4% formaldehyde/PBS for 15 min at room temperature. The fixative solution was removed and cells were washed three times with deionized water. The water was removed and 500 μl of 40 mM Alizarin Red S dye was added per well. Cells were incubated at room temperature for 30 min with gentle shaking. The dye was removed and cells were washed five times with deionized water before taking images. For tissue transplants, the sections were deparaffinized and hydrated to 70% ethanol. Then, the tissue sections were fixed with 10% formaldehyde/PBS for 15 min at room temperature before staining following the protocol described above.

### Real-Time PCR Analysis

Dental pulp stem cells were extracted for total RNA by using the RNeasy Mini kit (Qiagen) according to the manufacturer’s protocol. Quantity and purity of RNA was determined by 260/280 nm absorbance. First-strand cDNA was synthesized from 500 ng of RNA using the High Capacity cDNA synthesis kit (Applied Biosystems) per manufacturer’s protocols using a randomized primer. Real-time PCR primers are included in Supplementary Table 2.

A 20 ng of cDNA for Q-RT-PCR were prepared using the SYBR green PCR master mix (Applied Biosystems). Reactions were processed by the ABI 7900HT PCR system with the following parameters: 50°C/2 min and 95°C/10 min, followed by 40 cycles of 95°C/15 s and 60°C/1 min. Results were analyzed using SDS 2.2 software and relative expression calculated using the comparative Ct method. The threshold cycle (Ct) value for each gene was normalized to the Ct value of GAPDH. The relative mRNA expression, presented as fold change difference, was calculated by the comparative Ct method according to the formula: 2^–ΔΔCt^, where ΔCt = Ct target–Ct GAPDH and ΔΔCt = ΔCt target–ΔCt calibrator. Each sample was run in triplicate reactions for each gene. Error bars represent the standard deviation calculated from the triplicate analysis of each sample.

### *In vivo* Transplantation of DPSCs

In accordance with approved IACUC protocols, 1 × 10^6^ of VEGFR-2 shRNA treated DPSCs and CopGFP treated DPSCs were separately transplanted into 1-month-old male *Rag1* null mice (Jackson Laboratory, Bar Harbor, ME, United States) by dorsal subcutaneous transplantation with hydroxyapatite tricalciumphosphate (HAp/TCP) (Zimmer) (*n* = 5 mice/cell type). Grafts were harvested after 5 weeks of transplantation. Transplanted tissues samples were fixed with 4% formaldehyde for 2 h then demineralized for 7 days in 10% EDTA at 4°C. Then, the transplants were embedded in paraffin and cut to 5 μm thick sections. Sections were analyzed by H&E, Alizarin Red S and immunohistochemistry staining.

### Tissue Staining

Prior to staining procedures, paraffin-embedded tissue sections were deparaffinized and hydrated to 70% ethanol. Tissues were stained with H&E staining following the manufacturer’s standard protocol. For immunohistochemistry, fixed tissue sections were permeabilized with 1% BSA in 0.1% Triton-X 100 (Sigma)/PBS for 10 min, inhibited endogenous peroxidase activity with 0.3% hydrogen peroxide in methanol for 30 min, and blocked non-specific binding sites with 10% goat or horse serum (Vector Laboratories Inc., Burlingame, CA, United States) for 1 h. Primary antibodies listed in Supplementary Table 1 were used and incubated overnight at 4°C. Stained tissues were incubated with a biotinylated antibody at 1:100 (Vector Laboratories Inc., Burlingame, CA, United States) for 1 h, washed and treated with the Vectastain ABC kit and 3, 3′-diaminobenzidine (DAB) substrate kit according to manufacturer’s protocol (Vector Laboratories Inc., Burlingame, CA, United States).

### Statistical Analysis

The number of mice were calculated using power analysis based on previous pilot studies in our laboratory (statistical power of 0.90; *p* level = 0.05). Statistical analysis of the *in vitro* studies, histology, and molecular analysis results from three independent experiments were performed by the Student’s *t* test. Data are presented as mean ± SD. *p*-values ≤ 0.001, 0.005, 0.05 was considered as statistically significant.

## Results

### DPSCs Exhibited Self-Renewal and Multi-Differentiation Capacity *in vitro*

**FIGURE 1:**
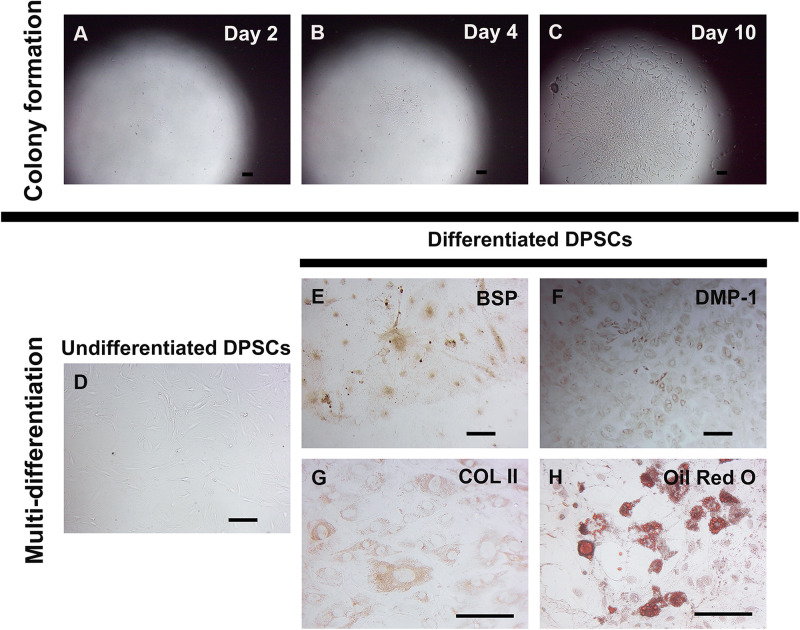
Self-renewal and *in vitro* multi-differentiation of murine DPSCs. Murine DPSCs were isolated from dental pulp of neonatal lower molar teeth. A low density of cells (1000 cells/cm^2^) were plated for several single cell depositions to observe a colony forming ability and characterize for their multi-differentiation. Representative figures show at the same spot of DPSC clone at different time points, Day 2 **(A)**, Day 4 **(B)**, and Day 10 **(C)**. The undifferentiated DPSC clone proliferated to gain the larger size, indicating its self-renewal property. The *in vitro* differentiation in various specific differentiation media demonstrates DPSC multi-differentiation potential. Undifferentiated DPSCs cultured in the stem cell media were spindle-shaped, indicating mesenchymal-like stem cell morphology **(D)**. Differentiated DPSCs cultured in the osteo-odontogenic media exhibited positive anti-BSP (in brown) **(E)** and anti-DMP-1 (in brown) **(F)** staining. Cultured in the chondrogenic media, differentiated DPSCs showed positive anti-COL II (in brown) **(G)** staining. Strongly positive Oil Red O staining (in red) **(H)** was seen in DPSC-derived lipid-containing cells in the adipogenic media. Scale bars indicate 100 μm.

Dental pulp stem cells were isolated from neonatal murine pulp tissue of lower molar teeth. Cell suspension was plated in a low cell density as a single cell deposition. After 2 days of culture in stem cell media, several cell colonies were observed (Figure 1A). Later, larger colonies of cells were seen before cell trypsinization and expansion (Figures 1B,C). Compared to undifferentiated DPSCs (Figure 1D), cultures in specific differentiation media revealed DPSCs differentiation abilities to give rise to osteoblast-like cells (positive staining for BSP and DMP-1, Figures 1E,F), chondrocyte-like cells (positive staining for COL II, Figure 1G), and adipocytes (positive staining with Oil Red O, Figure 1H).

### Down-Regulation of VEGFR-2 Significantly Decreases Expression of VEGF-A in DPSCs

**FIGURE 2:**
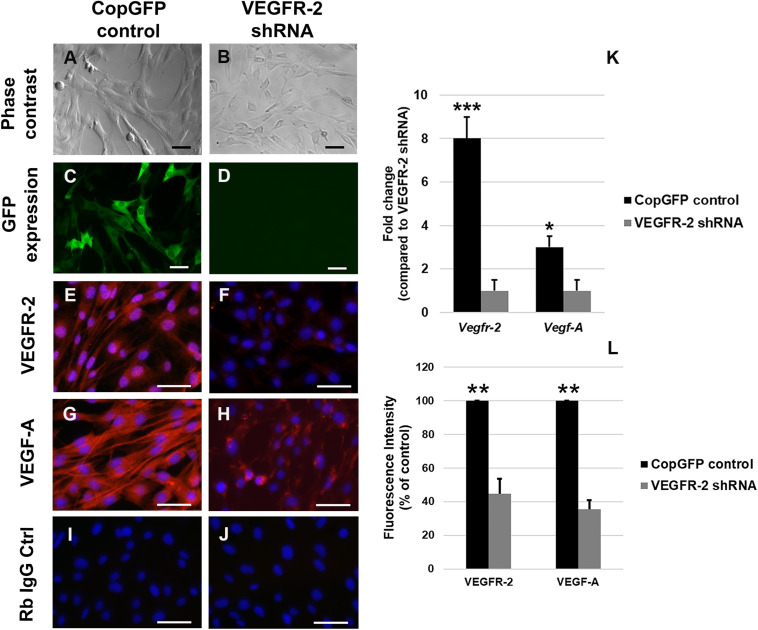
Silencing of VEGFR-2 in murine DPSCs using the lentiviral vector. Murine DPSCs were transfected by either CopGFP control **(A)** or VEGFR-2 shRNA **(B)** lentiviral vectors with 10:1 of MOI (multiplicity of infection). After transfection, both groups exhibited their regular spindle shapes, presenting no toxicity of the viral vector. The 100% expression of GFP in CopGFP control **(C)** but not in VEGFR-2 shRNA group **(D)** was seen. The GFP+ expression in CopGFP DPSCs indicates the high efficiency of transfection. Immunofluorescence (in red) demonstrates strong positive staining of VEGFR-2 **(E)** and its ligand, VEGF-A **(G)** in CopGFP control DPSCs whereas there were very low expression of VEGFR-2 **(F)** and VEGF-A **(H)** in VEGFR-2 shRNA DPSCs. The rabbit IgG control (Rb IgG Ctrl) stained in CopGFP control **(I)** and VEGFR-2 shRNA DPSCs **(J)** were completely negative to confirm the specificity of antibodies. DAPI (in blue) was used for the nuclear staining. Scale bars indicate 100 μm. The bar graph **(K)** reveals that real-time PCR analysis showed higher expression of both *Vegfr-2* and *Vegf-A* in CopGFP control DPSCs, compared to that in VEGFR-2 shRNA treated DPSCs. Differences in gene expression is demonstrated as the fold change of that in VEGFR-2 shRNA DPSC group. GAPDH was used as the internal control. The bar graph **(L)** reveals the quantification of the VEGFR-2 and VEGF-A fluorescence intensity in both normal and VEGFR-2 knockdown DPSCs by using ImageJ software. The quantification showed a statistically significant decrease in VEGFR-2 and VEGF-A levels in VEGFR-2 knockdown DPSCs, compared to normal DPSCs. The results were shown as the fluorescence intensity (% of control). ***, **, * is used for significantly statistical difference at *p*-value < 0.001, 0.005, 0.05, respectively. The results represent the means ± SD (*n* = 5 from each group/experiment) of three independent experiments.

In order to investigate the potential role of VEGFR-2 signaling in osteo-odontogenic differentiation of DPSCs, VEGFR-2 shRNA was utilized to silence VEGFR-2 expression in murine DPSCs. The CopGFP shRNA was also used as a control to determine the efficiency of transfection. We targeted DPSCs with both VEGFR-2 and CopGFP shRNA separately and observed approximately 100% GFP expression in CopGFP (Figures 2A,C) but not in VEGFR-2 shRNA group (Figures 2B,D). CopGFP DPSCs expressed high levels of both VEGFR-2 and VEGF-A (Figures 2E,G,K,L). In contrast, an 8-fold decrease of *Vegfr-2* expression was shown in VEGFR-2 shRNA DPSCs compared to the CopGFP control DPSCs (^∗∗∗^*p* < 0.001) (Figure 2K). The gene expression level corresponded to a dramatic decrease in VEGFR-2 immunofluorescence staining of VEGFR-2 shRNA DPSCs (Figures 2F,L). The quantification of the VEGFR-2 fluorescence intensity showed a statistically significant decrease in VEGFR-2 level in VEGFR-2 knockdown DPSCs, compared to normal DPSCs (^∗∗^*p* < 0.005) (Figure 2L). In addition, VEGFR-2 shRNA DPSCs down-regulated VEGF-A gene and protein levels (^∗^*p* < 0.05 and ^∗∗^*p* < 0.005, respectively), compared to the CopGFP control cells (Figures 2H,K,L). The rabbit IgG control (Rb IgG Ctrl) stained in CopGFP control (Figure 2I) and VEGFR-2 shRNA DPSCs (Figure 2J) were completely negative to confirm the specificity of antibodies.

### VEGFR-2 Deficient DPSCs Lacked Osteo-Odontogenic Potential *in vitro*

**FIGURE 3:**
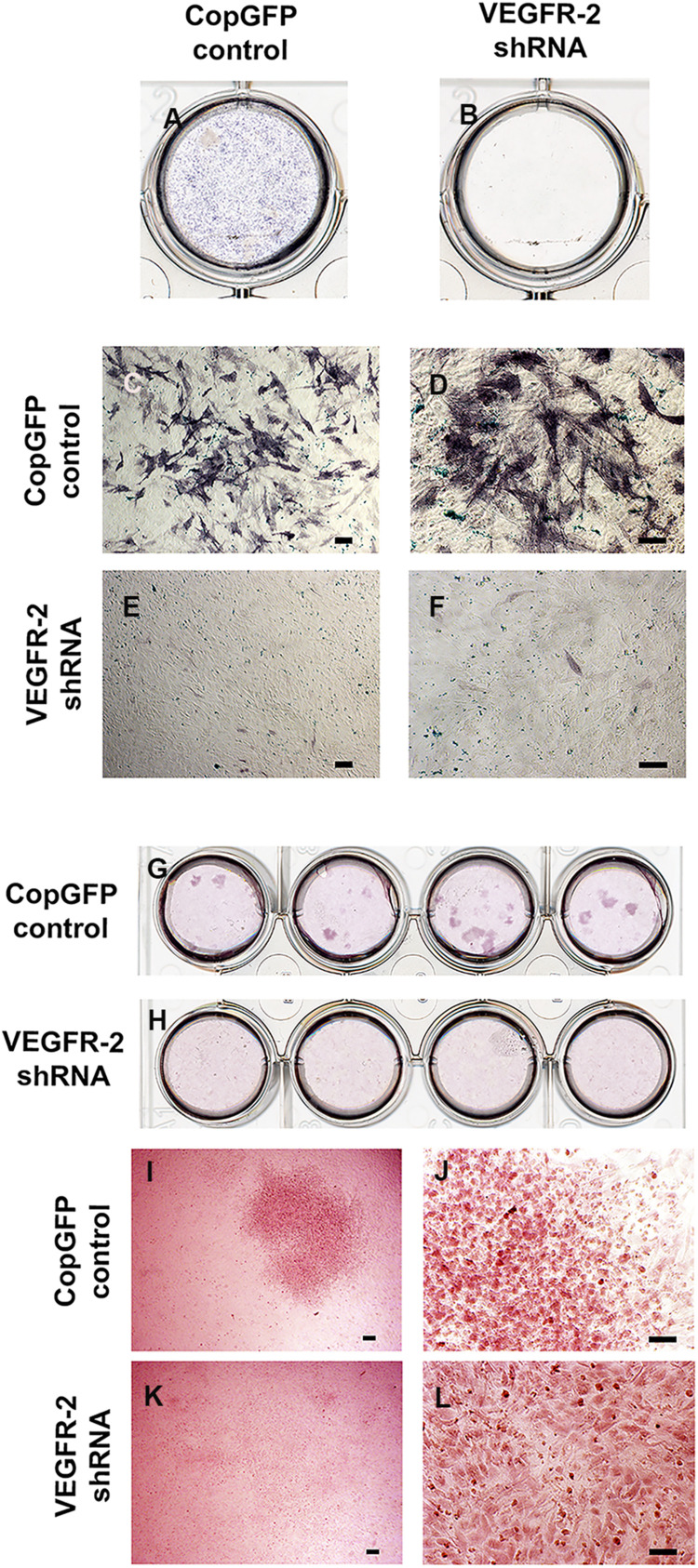
Alkaline phosphatase and Alizarin Red S staining in CopGFP control and VEGFR-2 shRNA DPSCs after a culture in osteo-odontogenic BMP-2 media. Alkaline phosphatase staining demonstrated a dissimilar result between CopGFP control **(A)** and VEGFR-2 shRNA DPSCs **(B)** after osteo-odontogenic induction in BMP-2 media. Abundant positive alkaline phosphatase stained cells (in dark-bluish purple) were shown in CopGFP control DPSCs, suggesting DPSCs undergoing osteo-odontogenesis **(C,D)**. Few slightly positive stained for alkaline phosphatase staining were found in VEGFR-2 shRNA DPSCs **(E,F)**. Alizarin Red S staining demonstrated a different result between CopGFP control and VEGFR-2 shRNA DPSCs after osteo-odontogenic induction in BMP-2 media. Several Alizarin Red S positive mineralized nodules were detected in differentiated CopGFP control DPSCs **(G)**. There was absence of mineralized nodule found in differentiated VEGFR-2 shRNA DPSCs **(H)**. A representative figure reveals a low magnification of Alizarin Red S positive mineralized nodules in the CopGFP control **(I)**. The higher magnification of the nodule showed cuboidal-like cells, resembling osteo-odontoblast-like cells **(J)**. VEGFR-2 shRNA DPSCs did not show any mineralized nodule formation **(K)** but they were undifferentiated spindle-shaped **(L)**. Scale bars indicate 100 μm.

**FIGURE 4:**
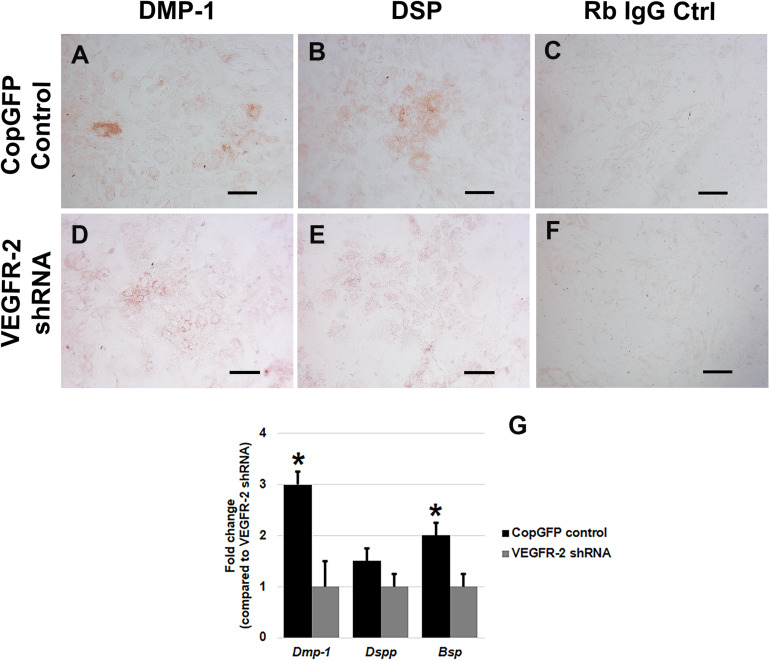
Dentin matrix protein staining and osteo-odontogenic gene expression in CopGFP control and VEGFR-2 shRNA DPSCs after a culture in osteo-odontogenic BMP-2 media. Immunocytochemistry showed certain positive cells of anti-DMP-1 **(A)** and anti-DSP **(B)** staining (in brown) in differentiated CopGFP control DPSCs but slightly stained cells in VEGFR-2 shRNA DPSCs **(D,E)**. The rabbit IgG control (Rb IgG Ctrl) stained in CopGFP control **(C)** and VEGFR-2 shRNA DPSCs **(F)** were completely negative to confirm the specificity of antibodies. Scale bars indicate 100 μm. The bar graph reveals that real-time PCR analysis showed higher expression of *Dmp-*1, *Dspp*, and *Bsp* in differentiated CopGFP control DPSCs, compared to that in differentiated VEGFR-2 shRNA treated DPSCs **(G)**. Differences in gene expression is demonstrated as the fold change of that in differentiated VEGFR-2 shRNA DPSC group. GAPDH was used as the internal control. * is used for significantly statistical difference at *p*-value < 0.05. The results represent the means ± SD of three independent experiments.

**FIGURE 5:**
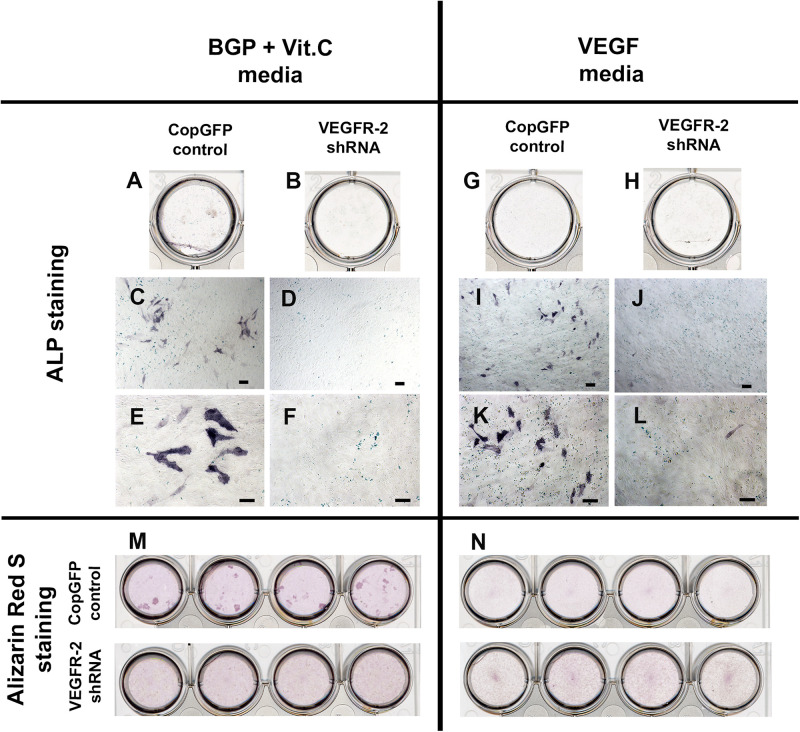
Alkaline phosphatase and Alizarin Red S staining in CopGFP control and VEGFR-2 shRNA DPSCs after a culture in BGP + Vit. C media and VEGF media. After induction in BGP plus vitamin C, some differentiated CopGFP DPSCs **(A,C,E)** but not VEGFR-2 shRNA DPSCs **(B,D,F)** demonstrated positive staining of alkaline phosphatase. Alizarin Red S staining showed some positive mineralized nodules in CopGFP DPSCs but not VEGFR-2 shRNA DPSCs **(M)**. For VEGF induction, a similar results of alkaline phosphatase staining were observed in both CopGFP control **(G,I,K)** and VEGFR-2 shRNA DPSCs **(H,J,L)**, compared to the BGP plus vitamin C. Interestingly, no mineralized nodule was found in both cell groups after VEGF induction **(N)**. Scale bars indicate 100 μm.

CopGFP DPSCs cultured in BMP-2 media showed strongly positive staining of ALP enzyme (in black) (Figures 3A,C,D). In contrast, VEGFR-2 shRNA DPSCs exhibited significantly decreased ALP staining (Figures 3B,E). Few slightly ALP positive cells in VEGFR-2 shRNA DPSCs were observed (Figure 3F). In addition, abundant mineralized nodules were observed and positively stained Alizarin Red S (in red) in the CopGFP DPSCs (Figures 3G,I) while those nodules were absent in the VEGFR-2 shRNA DPSCs (Figures 3H,K). A cluster of cuboidal cells were seen, indicating osteo-OLCs (Figure 3J). Nevertheless, Alizarin Red S stained slightly positive in the cytoplasmic compartment of VEGFR-2 shRNA DPSCs due to the intracellular calcium contents (Figure 3L). Remarkably, VEGFR-2 shRNA DPSCs maintained a spindle-shaped morphology, resembling an undifferentiated state. Additionally, immunocytochemistry showed certain positive cells of anti-DMP-1 (Figure 4A) and anti-DSP (Figure 4B) staining (in brown) in differentiated CopGFP control DPSCs but slightly stained cells in VEGFR-2 shRNA DPSCs (Figures 4D,E). The rabbit IgG control (Rb IgG Ctrl) stained in CopGFP control (Figure 4C) and VEGFR-2 shRNA DPSCs (Figure 4F) were completely negative to confirm the specificity of antibodies. VEGFR-2 shRNA DPSCs also down-regulated the expression of *Dmp-1*, *Dspp*, and *Bsp*; however, only *Dmp-1* and *Bsp* showed a significant difference compared to the CopGFP DPSCs (^∗^*p* < 0.05) (Figure 4G). Cultured in BGP + Vit. C media, occasional strongly ALP positive cells (Figures 5A,C,E) were seen in the control DPSCs but not in the VEGFR-2 shRNA DPSCs (Figures 5B,D,F). Some Alizarin Red S positive nodules were present in the CopGFP cells whereas those were not detected in the VEGFR-2 shRNA cells (Figure 5M). Similarly, cultured in VEGF media, few ALP positive cells were present in only the CopGFP group (Figures 5G,I,K), not in the VEGFR-2 shRNA group (Figures 5H,J,L). However, there were no mineralized nodules observed in both CopGFP and VEGFR-2 shRNA groups (Figure 5N).

### VEGFR-2 Deficient DPSCs Were Unable to Give Rise to Odontoblast-Like Cells and Form Pulp-Like Structures After Transplantation

**FIGURE 6:**
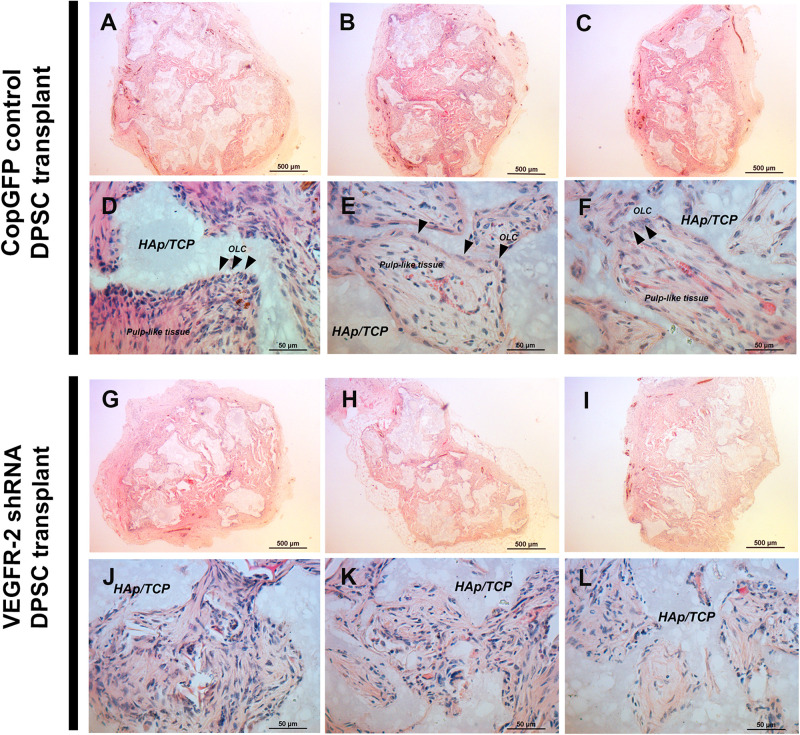
*In vivo* transplantation of CopGFP control and VEGFR-2 shRNA DPSCs by H&E staining. 1 × 10^6^ cells of CopGFP control and VEGFR-2 shRNA DPSCs were separately transplanted into 1-month-old male *Rag1* null mice by dorsal subcutaneous transplantation with hydroxyapatite tricalciumphosphate (HAp/TCP) (*n* = 5 mice per cell type). Transplanted tissues were harvested after 5 weeks, then proceeded and stained for analysis. H&E staining was performed to demonstrate the morphology of CopGFP DPSC transplants [**(A–C)**; low magnification of representative tissues] and VEGFR-2 shRNA DPSC transplants [**(G–I)**; low magnification of representative tissues]. Scale bars indicate 500 μm. CopGFP DPSC transplants exhibited elongated and polarized odontoblast-like cells (OLC) (arrowheads) and more organized loose connective tissue with abundant blood vessels mimicking pulp-like structures **(D–F)** while VEGFR-2 shRNA DPSC transplants showed an absence of odontoblast-like cells. Instead, disorganized fibrous collagenous tissue was seen **(J–L)**. OLC; odontoblast-like cells, HAp/TCP; hydroxyapatite/tricalciumphosphate. Scale bars indicate 50 μm.

H&E staining revealed the overall morphology of CopGFP DPSC transplants (Figures 6A–C) and VEGFR-2 shRNA DPSC transplants (Figures 6G–I). *In vivo* transplantation of CopGFP DPSCs demonstrated elongated and polarized cells (Figures 6D–F; arrowheads) running perpendicularly to hydroxyapatite/tricalcium phosphate (HAp/TCP), resembling the morphology of OLCs. In addition, a formation of pulp-like structure was revealed by the presence of loose connective tissue with capillaries containing RBCs (Figures 6D–F) in CopGFP DPSC transplants, mimicking pulp-like tissues in the natural teeth. In contrast, VEGFR-2 shRNA DPSCs transplants lacked OLCs and pulp-like structures. Instead, there was only a formation of disperse collagenous tissue (Figures 6J–L).

**FIGURE 7:**
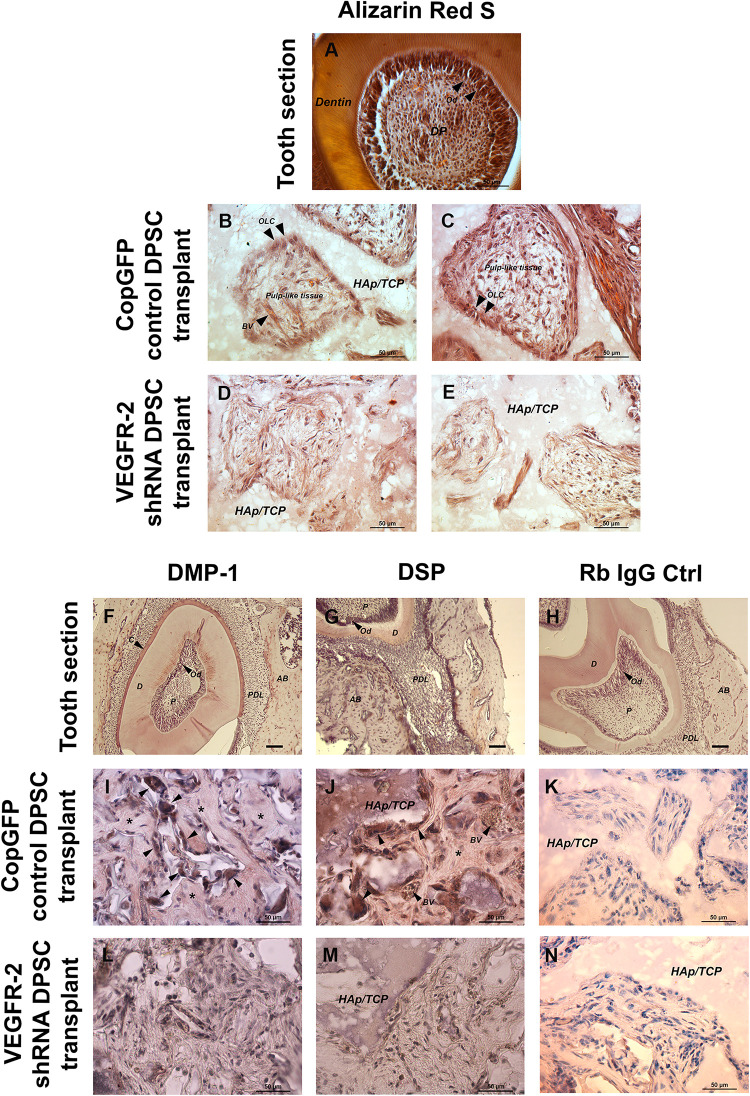
*In vivo* transplantation of CopGFP control and VEGFR-2 shRNA DPSCs by Alizarin Red S staining and immunohistochemistry. Alizarin Red S staining was conducted to confirm the mineralized tissues, dental structures and morphology of CopGFP DPSC transplants and VEGFR-2 shRNA DPSC transplants. The cross-section of murine tooth was used as a positive control for Alizarin Red S staining. The positive area of Alizarin Red S showed in dentin, odontoblasts and some dental pulp regions **(A)**. A CopGFP control DPSC transplant revealed positive staining of fibrous structure surrounding HAp/TCP with elongated and polarized odontoblast-like cells (OLC) (arrowheads). Some positive odontoblast-like cells (OLC) (arrowheads) running perpendicular to HAp/TCP and pulp-like tissues with blood capillaries were seen in CopGFP DPSC transplants **(B,C)**. VEGFR-2 shRNA DPSC transplants only showed diffuse unpatterned cell arrangement with disorganized connective tissue **(D,E)**. Immunohistochemistry of anti-DMP-1 and anti-DSP staining was also conducted to determine the specific dentin protein expression in the transplant tissues. The tooth sections were used as a positive control. The expression of DMP-1 and DSP was seen in dentin, cementum and alveolar bone. Both dentin matrices were very strong in the odontoblast cell layer **(F,G)**. The CopGFP control DPSC transplants revealed the strongly positive anti-DMP-1 and anti-DSP staining in odontoblast-like cells (OCL) (arrowheads) **(I,J)**. Its matrices were slightly stained for both markers (asterisks). The VEGFR-2 shRNA DPSC transplants showed the negative anti-DMP-1 and anti-DSP staining **(L,M)**. The Rabbit IgG control (Rb IgG Ctrl) was used as a negative control to confirm the specificity of antibody **(H,K,N)**. AB; alveolar bone, C; cementum, D; dentin, HAp/TCP; hydroxyapatite/tricalciumphosphate, OLC; odontoblast-like cells, P; Pulp, PDL; periodontal ligament. Scale bars indicate 50 μm.

In the cross-section of the tooth, Alizarin Red S showed the strongly positive staining of dentin and odontoblasts; it also exhibited the histology of dental pulp which is a vascularized loose connective tissue (Figure 7A). A transplanted tissue generated by the CopGFP DPSCs stained positive for Alizarin Red S and also demonstrated the presence of OLCs (Figures 7B,C; arrowheads) with pulp-like–tissues (Figures 7B,C). In contrast, VEGFR-2 shRNA DPSC transplants showed disorganized tissues faintly positive for Alizarin Red S staining (Figures 7D,E). In the tooth section, anti-DMP-1 and anti-DSP staining was positive in dentin and intense in odontoblasts (Figures 7F,G). Interestingly, the transplanted tissues generated by the control cells also showed strong signal of immunohistochemistry for DMP-1 and DSP proteins in OLCs (Figures 7I,J; arrowheads) and matrix (Figures 7I,J; asterisks). In contrast, we were not able to observe positive DMP-1 and DSP staining in the VEGFR-2 shRNA DPSC transplants (Figures 7L,M). Rb IgG control was used as a negative control (Figures 7H,K,N).

## Discussion

Dental pulp stem cells which are isolated from tooth pulp tissues have been shown to play an important role in dentin/pulp repair and regeneration (Gronthos et al., 2002; Paz et al., 2018). Recent studies have revealed that DPSCs have not only an odontogenic capacity but also an angiogenic potential (Martinez-Sarra et al., 2017; Yamada et al., 2019; Shi et al., 2020; Sui et al., 2020). We have previously shown that angiogenesis induced by DPSCs is regulated by VEGFR-2 signaling (Janebodin et al., 2013). However, the relevance of VEGFR-2 signaling in odontogenesis and dentinogenesis is still undetermined. In this study, we investigated the potential role of VEGFR-2 for odontogenic capacity of murine DPSCs both *in vitro* and *in vivo*.

First, DPSCs were isolated from mouse neonatal-derived lower molar teeth and examined for their stem cell properties as previous protocol (Janebodin et al., 2011). Plating cells at low density close to a single cell deposition revealed formation of several cell colonies. As expected, those colonies increased in size with longer culture time, highlighting their self-renewal capacity. Moreover, DPSCs cultured in various differentiation media exhibited characteristic morphological changes combined with the positive staining for osteoblastic, chondrogenic, and adipogenic. These results correspond to previous studies confirming the mesenchymal stem cell’s properties of DPSCs (Gronthos et al., 2000; Huang et al., 2008; Janebodin et al., 2011; Paz et al., 2018).

Next, we silenced the expression of VEGFR-2 in DPSCs by using the VEGFR-2 shRNA lentiviral transfection. The shRNA (short hairpin RNA) delivery into mammalian cells is considered a powerful method to study gene functions via the cellular mechanism of RNA interference. This technique provides a stable integration of shRNA, resulting in a long-term knock down of an interested gene (Moore et al., 2010). To confirm the initial successful transfection, treated cells were selected through puromycin supplemented media. Furthermore, the CopGFP shRNA which is a non-specific viral vector conjugated with GFP was used as a control (Guryanova et al., 2006). Approximately 100% of DPSCs transfected with the CopGFP construct expressed GFP. Since both CopGFP and VEGFR-2 shRNA have the similar constructs, we assumed that transfection of DPSCs with VEGFR-2 shRNA was highly efficient. To prove that, real-time PCR and immunofluorescence were used to demonstrate that the VEGFR-2 shRNA DPSCs exhibited a decrease in VEGFR-2 gene and protein expression, compared to the CopGFP control. Importantly, the transcriptional and translational level of VEGF-A were also down-regulated. VEGF-A is a protein with a variety of biological functions especially in endothelial cells and pericytes such as vascular permeability, cell survival, proliferation and migration that are important in angiogenesis. VEGF-A binds to and activates VEGFR-1 and VEGFR-2 (Neufeld et al., 1999). Although VEGFR-2 has a low affinity for VEGF-A, its tyrosine kinase activity is stronger than that of VEGFR-1 (Eichmann and Simons, 2012; Berendsen and Olsen, 2014). Previous studies have shown that DPSCs, a neural crest-derived mesenchymal stem cell, have pericyte-like properties capable of inducing angiogenesis through VEGFR-2/VEGF-A pathway (Janebodin et al., 2013). This suggests that the down-regulation of VEGFR-2 may regulate expression of its ligand, VEGF-A. Nevertheless, the underlying mechanism needs further investigation.

The osteo-odontogenic capacity of CopGFP and VEGFR-2 shRNA DPSCs were examined by cultured in two types of osteo-odontogenic media, BMP-2 media and BGP plus vitamin C media. The CopGFP control cells exhibited higher osteo-odontogenic potential in both media, compared to the VEGFR-2 shRNA cells based on ALP and Alizarin Red S staining. However, CopGFP cells cultured in BMP-2 media showed higher number of differentiated cells compared to those in BGP plus vitamin C media. This result underscores BMP-2 as a powerful morphogen in osteoblast differentiation, bone development, formation and repair (Jorgensen et al., 2004; Marupanthorn et al., 2017). ALP plays an important role as an early osteogenic marker (Sabokbar et al., 1994). Alizarin Red S staining is a method to determine the mineralization or calcification which is a late hallmark for osteoblastic differentiation (Clark et al., 2015). Additionally, osteo-odontogenic genes, *Dmp-1*, *Dspp*, and *Bsp*, were examined. Those markers have been previously reported as important factors for bone and dentin formation (Hwang et al., 2008; Ching et al., 2017). Following differentiation, the CopGFP cells expressed statistically higher levels of *Dmp-1*, *Dspp*, and *Bsp*, compared to the VEGFR-2 shRNA cells. This demonstrate that the VEGFR-2 shRNA DPSCs were unable to differentiate into the osteo-odontogenic lineage *in vitro*. Taken together, this suggests that VEGFR-2 may play an important role in osteo-odontogenic potential of DPSCs.

To further investigate whether VEGF-A signaling via VEGFR-2 directly induces odontoblastic differentiation of DPSCs, the CopGFP and VEGFR-2 shRNA DPSCs were cultured in VEGF media and evaluated by ALP and Alizarin Red S staining. A few CopGFP cells only demonstrated positive ALP staining; however, none of VEGFR-2 shRNA cells showed a positive result. Moreover, neither CopGFP cells nor VEGFR-2 shRNA cells exhibited the positive Alizarin Red S staining. This indicates that VEGF-A only cannot induce a complete and mature osteo-odontogenic differentiation. Instead, VEGF-A might facilitate or “prime” DPSCs for the early stages of differentiation but it seems insufficient on its own to induce full osteo-odontogenic differentiation. These results are consistent with previous studies showing that exogenous VEGF enhances proliferation and early differentiation of DPSCs (D’Alimonte et al., 2011; Forgues et al., 2019). Nevertheless, our current study helps us differentiate the role of VEGFR-2 in the osteo-odontogenic ability of DPSCs, independent of VEGF-A.

To confirm our *in vitro* differentiation, the CopGFP and VEGFR-2 shRNA DPSCs mixed with hydroxyapatite/tricalciumphosphate (HAp/TCP) were subcutaneously transplanted in the dorsum of Rag-1 knockout mice for 5 weeks. This knockout mouse model is immunodeficient due to an absence of B- and T-lymphocytes (Menoret et al., 2013), and thus a good model to avoid transplant rejection. The CopGFP DPSC transplants exhibited elongated and polarized OLCs with vascularized pulp-like structures, corresponding to several previous studies of DPSC transplants with osteoconductive HAp/TCP scaffolds (Gronthos et al., 2000, 2002). In contrast, the VEGFR-2 shRNA DPSC transplants showed only the formation of collagenous and fibrous tissues. Consequently, the CopGFP DPSC transplantation confirms a biological role of VEGFR-2 for DPSC odontogenic potential.

In addition to endothelial cells, VEGF signaling has been shown to exhibit an important function in osteoblasts (Deckers et al., 2000). Previous *in vitro* experiments suggested that VEGFR-2 plays a role in osteoblast differentiation and survival (Alonso et al., 2008). Recently, the role of VEGF signaling and intracellular VEGF in osteoblast differentiation have been reported (Liu et al., 2012). BMP-induced VEGF expression is an important mechanism during osteoblast differentiation and bone formation (Zelzer and Olsen, 2005). Unlike the osteoblast differentiation, the role of VEGFR-2 in odontoblastic differentiation of DPSCs is still undetermined. Our data showed that VEGFR-2 shRNA DPSCs cultured in BMP-2 media were unable to give rise into the osteo-odontogenic lineage. One possibility is that VEGFR2 signaling is necessary for up-regulation of BMP-2 receptors (BRII and BRII) and downstream signaling molecules (Smads), thus priming odontoblast precursors to be responsive to BMP-2 (Qin et al., 2012). Additionally, silencing VEGFR-2 in DPSCs led to reductions in VEGF-A levels. Therefore, impaired odontoblast differentiation of VEGFR-2 null cells may be due to synergist or independent effects of VEGF-A.

However, our studies showed that adding exogenous VEGF to treat CopGFP and VEGFR-2 shRNA cells did not induce complete odontogenic differentiation. This is corresponding to a previous study showing that treatment with recombinant VEGF does not affect osteoblastic and adipocytic differentiation in normal bone marrow derived MSCs and VEGF deficient MSCs (Liu et al., 2012). Altogether, this suggests the effects of VEGFR-2 are mediated by intracellular mechanisms, possibly including transcriptional regulation of BMP signaling proteins rather than by paracrine VEGF signaling. In addition to intracrine mechanisms, it is possible that DPSC-derived VEGF also affects the formation of dentin/pulp complex *in vivo* through enhanced angiogenesis (Wang et al., 2007). Nonetheless, the specific mechanism(s) of how VEGFR-2 affects odontoblast differentiation of DPSCs need to be further investigated.

In conclusion, our data originally identifies VEGFR-2 signaling as an important molecular pathway for odontoblastic differentiation in DPSCs. These results provide key evidence of the importance of VEGFR-2 in tooth development and highlight the potential of VEGFR-2 modulation to enhance dentin regeneration and tissue engineering as a promising clinical application such as targeted drug therapy and delivery for regenerative dentistry.

## Data Availability Statement

The raw data supporting the conclusions of this article will be made available by the authors, without undue reservation.

## Ethics Statement

The animal study was reviewed and approved by University of Washington.

## Author Contributions

KJ and MR: writing and editing and study design. KJ, RC, and AH: experimentation. KJ, RC, and MR: data analysis. All authors contributed to the article and approved the submitted version.

## Conflict of Interest

The authors declare that the research was conducted in the absence of any commercial or financial relationships that could be construed as a potential conflict of interest.
