# Supplementary material for: Silencing VEGFR-2 Hampers Odontoblastic Differentiation of Dental Pulp Stem Cells
Source: Front Cell Dev Biol. 2021 Jun 25;9:665886. doi: 10.3389/fcell.2021.665886 (PMC8267829; doi:10.3389/fcell.2021.665886)
Supplement: Supplementary file 1 [file Table_1.docx]

**Supplementary Table 1. Lists of antibodies for staining**

| **Marker** | **Antibody** | **Species** | **Dilution** | **Company** |
| --- | --- | --- | --- | --- |
| BSP | Monoclonal | Mouse | 1:100 | Developmental Hybridoma Bank |
| COLII | Monoclonal | Mouse | Whole supernatant | Developmental Hybridoma Bank |
| DMP-1 | Polyclonal | Rabbit | 1:400 | Takara |
| DSP | Polyclonal | Rabbit | 1:200 | Larry Fisher, NIDCR/NIH |
| VEGFR-2 | Polyclonal | Rabbit | 1:100 | ECM Bioscience |
| VEGF-A | Polyclonal | Rabbit | 1:100 | Abcam |
