# Supplementary material for: Silencing VEGFR-2 Hampers Odontoblastic Differentiation of Dental Pulp Stem Cells
Source: Front Cell Dev Biol. 2021 Jun 25;9:665886. doi: 10.3389/fcell.2021.665886 (PMC8267829; doi:10.3389/fcell.2021.665886)
Supplement: Supplementary file 2 [file Table_2.docx]

| **Gene** | **Forward primer**  **(5’🡪3’)** | **Reverse primer**  **(5’🡪3’)** | **GenBank Accession number** | **Product size (bps)** |
| --- | --- | --- | --- | --- |
| *Vegfr2* | ACTGCAGTGATTGCCATGTTCT | CCTTCATTGGCCCGCTTAA | NM_010612 | 74 |
| *Vegfa* | GAGCAGAAGTCCCATGAAGTGAT | CAATCGGACGGCAGTAGCTT | NM_001025250 | 67 |
| *Dmp-1* | AGTGAGTCATCAGAAGAAAGTCAAGC | CTATACTGGCCTCTGTCGTAGCC | NM_016779 | 107 |
| *Dspp* | AACTCTGTGGCTGTGCCTCT | TATTGACTCGGAGCCATTCC | NM_010080 | 171 |
| *Bsp* | TGGTGCTACAGCCCCAGAGT | AAAGAAGAAGCCCACTACAAACAAA | NM_008318 | 61 |
| *Gapdh* | GGGAAGCCCATCACCATCT | GCCTCACCCCATTTGATGTT | NM_008084 | 59 |

**Supplementary Table 2. The mouse-specific primer sequences**
